# Distributed Strain Monitoring Using Nanocomposite Paint Sensing Meshes

**DOI:** 10.3390/s22030812

**Published:** 2022-01-21

**Authors:** Sijia Li, Yening Shu, Yun-An Lin, Yingjun Zhao, Yi-Jui Yeh, Wei-Hung Chiang, Kenneth J. Loh

**Affiliations:** 1 Department of Structural Engineering, University of California San Diego, La Jolla, San Diego, CA 92093-0085, USA; sil024@ucsd.edu (S.L.); yeshu@eng.ucsd.edu (Y.S.); yul095@eng.ucsd.edu (Y.-A.L.); irenezhao@utexas.edu (Y.Z.); 2 Active, Responsive, Multifunctional, and Ordered-Materials Research (ARMOR) Laboratory, La Jolla, San Diego, CA 92093-0085, USA; 3 Department of Chemical Engineering, National Taiwan University of Science and Technology, Taipei City 106, Taiwan; yaeraser@gmail.com (Y.-J.Y.); whchiang@mail.ntust.edu.tw (W.-H.C.)

**Keywords:** carbon nanotube, EIT, electrical resistance tomography, ERT, sensor, strain field, structural health monitoring, thin film

## Abstract

Strain measurements are vital for monitoring the load-bearing capacity and safety of structures. A common approach is to affix strain gages onto structural surfaces. On the other hand, most aerospace, automotive, civil, and mechanical structures are painted and coated, often with many layers, prior to their deployment. There is an opportunity to design smart and multifunctional paints that can be directly pre-applied onto structural surfaces to serve as a sensing layer among their other layers of functional paints. Therefore, the objective of this study was to design a strain-sensitive paint that can be used for structural monitoring. Carbon nanotubes (CNT) were dispersed in paint by high-speed shear mixing, while paint thinner was employed for adjusting the formulation’s viscosity and nanomaterial concentration. The study started with the design and fabrication of the CNT-based paint. Then, the nanocomposite paint’s electromechanical properties and its sensitivity to applied strains were characterized. Third, the nanocomposite paint was spray-coated onto patterned substrates to form “Sensing Meshes” for distributed strain monitoring. An electrical resistance tomography (ERT) measurement strategy and algorithm were utilized for reconstructing the conductivity distribution of the Sensing Meshes, where the magnitude of conductivity (or resistivity) corresponded to the magnitude of strain, while strain directionality was determined based on the strut direction in the mesh.

## 1. Introduction

Structures in the civil, mechanical, and aerospace industries deteriorate over time and can possibly result in unexpected failure when exposed to extreme loads. Moreover, hazardous environmental conditions and a lack of maintenance can accelerate structural degradation, significantly shortening structural service lifetimes and causing catastrophic loss. Structural health monitoring (SHM) provides techniques to streamline the measurement of real-time structural performance data [1], which can be used to identify structural degradation and provide maintenance guidance so that early signs of damage prior to structural failure can be flagged.

Performing stress analysis on an existing structure can be an effective way to estimate its remaining service life, thereby preventing catastrophic structural failure [2]. However, stress states are not directly measurable. However, they can be correlated from the strain states of a known material or structure. The strain state of a structure can be directly assessed in situ via instrumenting various kinds of strain transducers [3], and the collected data can be used for damage localization and life-cycle analysis.

The most widely used strain transducer is a foil-based strain gage that can be directly affixed onto the surface of the structure of interest [4]. Foil-based strain gages have many advantages, including high accuracy, low cost, stable performance, and an easy installation process. However, they are discrete sensors that can only measure strains at the point of installation. In practice, high-precision data collection of large civil infrastructures requires installing dense sensor arrays. Another approach, as described by Yao et al. [5], is to fabricate a strain sensing sheet with a dense array of strain gages connected with full circuits. Zymelka et al. [6] developed an array of 25 printed carbon-based strain gages equipped with a wireless data acquisition system for long-term strain monitoring. However, sensor arrays can still only measure discrete strains, and they can be expensive and challenging to implement. Alternatively, optical-fiber-based strain transducers, such as the fiber Bragg grating (FBG) strain sensor, may be used to measure 1D strain distribution along its fiber length. FBGs outperforms other similar instruments due to their high strain sensitivity, immunity to electromagnetic noise, and deformation sensitivity [7,8]. However, their high fabrication and installation costs, as well as the requirement of complex data acquisition systems, have limited their use for high-value capital projects.

Recent developments in nanotechnology have inspired assembling multifunctional nanocomposite coating materials as strain sensors for SHM applications [9]. Multifunctional nanocomposite coatings are thin films assembled using a bottom-up design methodology to meet specific engineering design criteria. Among many of the available functionalized nanomaterials, carbon nanotubes (CNT) possess outstanding mechanical and electrical properties that can be utilized for fabricating strain-sensitive (i.e., piezoresistive) thin films [10,11]. For example, Dai et al. [12] proposed a method to apply CNT-based piezoresistive composite coatings over unwoven fibers as strain sensors and observed a linear piezoresistive response of the coating subjected to elastic tensile and compressive strains. Dharap et al. [13] fabricated a single-walled carbon nanotube buckypaper strain sensor and found that its voltage response varied linearly with respect to the applied strains. Loh et al. [14] employed the layer-by-layer method to assemble CNT-polyelectrolyte thin films with uniform thickness and a higher strain-sensing range.

Many studies have extended the application of CNT thin films from point-based strain gages to distributed strain sensors. Hou et al. [15] successfully coupled CNT-based thin films with electrical impedance tomography (EIT) for spatial stimuli sensing. Loh et al. [16] applied CNT-based sensing skin onto a metallic plate to achieve spatial impact damage identification. Hou and Lynch [17] correlated the change in EIT conductivity distributions to applied strains and found a linear relationship. Loyola et al. [18,19] implemented CNT-based thin films between the plies of a glass fiber–reinforced polymer (GFRP) composites and reconstructed the EIT conductivity changes to identify the subsurface impact damage. Some studies have also examined other conductive coatings coupled with EIT to achieve spatial sensing. Hallaji et al. [20,21] coated a layer of conductive silver paint onto a concrete beam and performed EIT analysis to identify shear crack initiation and crack growth during three-point bending. Tallman et al. [22] used the difference imaging algorithm to perform EIT conductivity reconstruction of a carbon black filled glass fiber/epoxy laminate to detect the plastic zone growth of a center crack.

Previous works have demonstrated EIT’s capability toward identifying the location and severity of damage in a conductive body, and CNT-based nanocomposite coatings may functionalize the structure to be interrogated. Whereas strain magnitudes can be estimated, strain fields (i.e., magnitudes and directionalities) cannot be reconstructed using classical EIT. To address this limitation, a “sensing mesh” composed of directional elements by patterning a nanocomposite coating was developed [23]. This approach was to configure the nanocomposite coating to form a grid-like pattern so that the strain magnitude along each directional element can be reconstructed from EIT, while their directions were based on the longitudinal axes of the grid’s struts.

This study begins with introducing the mathematical background of the EIT algorithm, followed by the experimental details on nanocomposite coating fabrication and testing. A commercially available vinyl-based paint was used as the matrix material to design the CNT-based nanocomposite paint. The optimal CNT concentration and paint formulation were determined based on the physical and electrical properties of the dried nanocomposite paint. Then, the nanocomposite paint was airbrushed onto a set of 3D-printed tensile testing coupons that were then subjected to uniaxial tensile cyclic tests for characterizing its electromechanical properties. Upon confirming that the nanocomposite coating’s strain sensitive behavior, sensing meshes were fabricated by coating patterned grid-like substrates. The sensing mesh specimens were subjected to different types of loads for distributed strain sensing validation. The experimental results and plans for future work are discussed at the end.

## 2. Background: Electrical Impedance Tomography

Electrical impedance tomography (EIT) is a technique that reconstructs the two-dimensional (2D) or three-dimensional (3D) conductivity distribution of a conductive body with a known boundary [16]. Originally proposed as a biomedical imaging technique [24], EIT has gained popularity and has been adapted for SHM. In order to generate EIT reconstruction of conductivity change, the forward and inverse problems need to be solved [25].

### 2.1. Forward Problem

If the electrical conductivity distribution (*σ*) of a conductive body (Ω) is known, then the electrical boundary potential (*u*) can be calculated by solving the EIT forward problem. Mathematically, the EIT forward problem is expressed as the 2D Laplace equation, which is derived from Maxwell’s equations [26]:

(1) $$\nabla \cdot \left(\sigma \nabla u\right)=0,$$

In general, it is difficult to solve the EIT forward problem in the continuum domain, since a continuum conductivity distribution function and continuum applied boundary current function are practically unobtainable [16]. Instead, the domain can be discretized into linear triangular elements using a finite element model (FEM) [27]. This study implemented a grid-like patterned sensing mesh instead of using a continuous conductive thin film. Therefore, uniaxial truss elements that represented each struct in the patterned sensing mesh were employed. In doing so, the computational demand to solve the forward problem was reduced significantly.

In general, the forward problem of the FEM system can be expressed as the linear equation [25,26,28]:

(2) $$\left[\begin{array}{cc} A_{M}+A_{Z} & A_{W}\\ A_{W}^{T} & A_{D} \end{array}\right]\left[\begin{array}{c} u\\ V \end{array}\right]=\left[\begin{array}{c} 0\\ I \end{array}\right],$$

where

(3) $${\left[A_{M}\right]}_{ij}=\int_{\Omega }^{}\sigma \nabla \varphi_{i}\cdot \nabla \varphi_{j}dxdy,$$

(4) $${\left[A_{Z}\right]}_{ij}=\overset{L}{\underset{l=1}{\sum }}\int_{E_{l}}\frac{1}{z_{l}}\varphi_{i}\varphi_{j}dS,$$

(5) $${\left[A_{W}\right]}_{i}=-\frac{1}{z_{l}}\int_{E_{l}}\varphi_{i}dS,$$

(6) $$\left[A_{D}\right]=diag\left(\frac{\left|E_{l}\right|}{z_{l}}\right),$$

The unknown nodal potentials at each node (***u***) and boundary electrode voltage (***V***) can be solved using the known current injections (***I***) applied to the corresponding electrode pairs. In Equations (3)–(5), *φ*_i_/*φ*_j_ represents the *i*th/*j*th finite element linear basic function, where *i*,*j* = 1, 2, …, N nodes. *Z_l_* is the contact impedance between the *l*th electrode and the domain, and *E_l_* is the length of the *l*th electrode. The standard diffusion stiffness matrix [*A*_M_] is an N × N symmetric matrix that represents the usual system matrix for the governing equation of the numerally meshed domain [25], while [*A*_Z_], [*A*_W_], and [*A*_D_] implement the boundary conditions. More details about the EIT forward problem have been discussed by Kaipio et al. [29].

### 2.2. Inverse Problem

In practical applications of EIT, the inverse problem needs to be solved to estimate or reconstruct the conductivity distribution of the target. Specifically, EIT uses boundary current excitations and voltage measurements to reconstruct the conductivity distribution within the boundary. To collect voltage responses, a set of electrodes should be installed along the boundary of the body, and a direct or alternative current (DC or AC) is applied to a pair of electrodes. In the case of using DC excitations, the EIT problem specializes to become electrical resistance tomography (ERT). Regardless, the applied electrical current can cause the development of boundary potential across the remaining electrodes pairs, and voltage across different pairs of remaining boundary electrodes are measured. This excitation process is repeated until all the electrodes pairs have been injected with current.

In this work, the Maximum a posteriori (MAP) reconstruction algorithm first developed by Alder and Guardo [30] was used to solve the ERT inverse problem. MAP is a one-step linearization solver, which is ideal for real-time measurements [19]. The normalized change in electrical conductivity (i.e., conductivity change divided by initial conductivity, *σ*_0_) between the intact and damaged condition of the conductive domain following the regularized inverse as [18,19,25,30,31]:

(7) $$\left\{\left.\frac{\Delta \sigma }{\sigma_{0}}\right\}\right.=\left[{\left(H^{T}WH+\lambda R\right)}^{-1}H^{T}W\right]\left\{\left.\frac{\Delta V}{V_{0}}\right\}\right.,$$

where

(8) $${\left[H\right]}_{ij}=-\int \sum_{i=1}^{2}{\left(\nabla u\right)}_{i}{\left(\nabla u^{*}\right)}_{j}dxdy,$$

(9) $$W_{i,i}=\frac{1}{\alpha_{i}}.$$

[*H*] is the sensitivity matrix that correlates a small normalized change in conductivity in one element to a normalized change in boundary electrodes voltage [18]. The covariance matrix ([*W*]) responds to the Gaussian white noise from every voltage measurement, where variable *α_i_* is the variance of the corresponding *i*th measurement [19]. The regularization matrix ([*R*]) implements the condition of smoothing and stabilization (e.g., NOSER [32], Tikhonov’s [33], Gaussian high-pass filter [30], and Laplacian filter [34]), and λ is the regularization hyperparameter that controls the amplification of noise in the reconstruction images [25]. To perform the reconstruction, two sets of boundary electrode voltage measurements are required, where a baseline voltage measurement set (*V*_0_) is first obtained, followed by another set that corresponds to a different state [19].

The regularization hyperparameter (*λ*) must be chosen to maintain a balance between excluding data errors caused by noise while still adequately fitting the experimental data, which can be accomplished by the noise figure (NF) of the MAP algorithm. NF is defined as the signal-to-noise ratio (SNR) of the voltage SNR (SNR*_V_*) divided by the reconstructed conductivity SNR (SNR*_σ_*) [35]. More detailed descriptions of the SNR calculations can be found in other published works [25,30,35]. In this study, the goal was to update the value of λ until NF was equal to 1. This value was selected to prevent the reconstruction from being over- or under-smoothed so that the difference between the SNR*_V_* of experimentally measured boundary voltages and the SNR*_σ_* of reconstructed conductivity would be minimized. After determining the regularization hyperparameter, the reconstructed conductivity change of the sensing domain can be performed using the MAP linear reconstruction equation.

## 3. Experimental Details

### 3.1. Nanocomposite Paint

#### 3.1.1. Materials

Pro-Line vinyl copper antifouling paint and Pro-Line vinyl paint thinner from Sherwin-Williams (Garland, TX, USA) were used in this study. Multi-walled carbon nanotubes (MWCNT) with an outer diameter of 8 nm were purchased from NanoIntegris (Boisbriand, QC, Canada), and carbon black (CB) SC159 was provided by Tokai Carbon CB (Fort Worth, TX, USA). For the substrates, thermoplastic polyurethane (TPU) sheet was acquired from Wiman Corporation (Sauk Rapids, MN, USA), a clear polyvinyl chloride (PVC) plate was ordered from McMaster-Carr (Santa Fe Springs, CA, USA), and strain gages were purchased from Tokyo Sokki Kenkyujo (Tokyo, Japan). For the electrodes, colloidal silver paste was from Ted Pella (Redding, CA, USA), two-part silver epoxy was from MG Chemicals (Surrey, BC, Canada), solder wire was purchased from Voltera (Kitchener, ON, Canada), and self-adhesive copper tape was from 3M (Maplewood, MN, USA).

#### 3.1.2. Spray Coating

**Figure 1:**
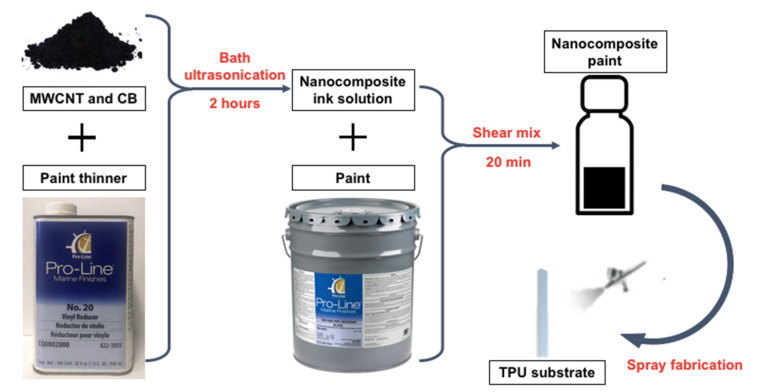
The CNT-CB nanocomposite paint preparation and thin film spray-coating process are illustrated.

The sprayable, strain-sensitive nanocomposite paint was prepared by first mixing MWCNT and CB with the paint thinner. Then, the mixer was subjected to bath ultrasonication for 2 h to disperse the MWCNTs and CBs. The resulting dispersion is referred to as the ink. After obtaining the nanocomposite ink, vinyl-based paint (which is too viscous to for direct use) was added to the ink and shear-mixed for 20 min at 1000 rpm to obtain the final nanocomposite paint. The nanocomposite paint was then directly spray-coated by hand, using a Paasche airbrush, onto the rough side of the TPU sheet substrate. The fabrication process is illustrated in Figure 1, and films can be deposited within minutes when using spray coating. The last step entailed air-drying the specimens at ambient room temperature for at least 12 h prior to their use.

### 3.2. Strain Sensing Characterization

**Figure 2:**
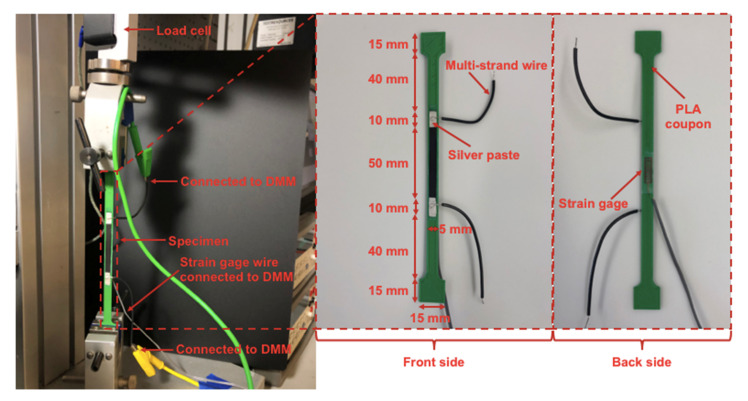
Nanocomposite paint was spray-coated onto a TPU substrate and then affixed onto a PLA test coupon. A strain gage was mounted onto the backside of the coupon and subjected to electromechanical tests.

The strain sensing properties of nanocomposite paint specimens were characterized using paint spray-coated onto 5 × 70 mm^2^ rectangular TPU substrates. After the nanocomposite paint dried, multi-strand wires were soldered onto copper tape using Voltera solder wire, and these electrodes were affixed onto the two opposite ends of each specimen. Colloidal silver paste dabs were also applied over the copper tape and nanocomposite paint to reduce contact impedance. Each rectangular nanocomposite paint specimen was then affixed onto a 3-D printed dog bone-shaped polylactic acid (PLA) coupon. A metal-foil strain gage (gage factor = 2.12 ± 1%) was also mounted onto the backside of the PLA coupon to obtain reference strain measurements. Then, the coupon was mounted in a Test Resources 150R load frame (Figure 2) and subjected to cyclic tensile loads (peak displacement of 0.09 mm at a constant load rate of 0.03 mm/s). During testing, the electrical resistance of the nanocomposite paint, as well as the strain gage, were recorded simultaneously using two Keysight 34450A digital multimeters (DMM) and the *BenchVue* data logging software.

### 3.3. Sensing Mesh ERT Validation

**Figure 3:**
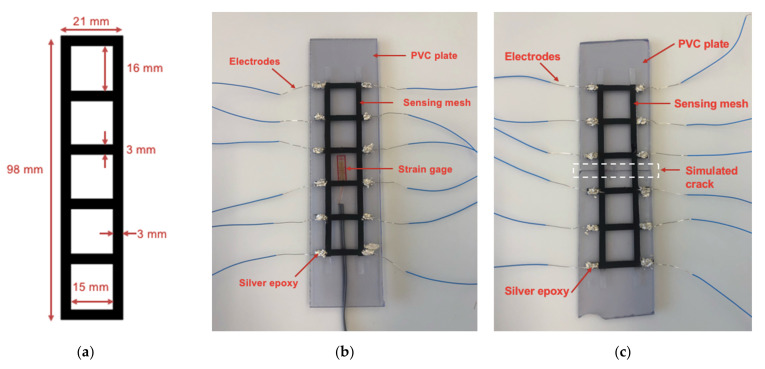
(**a**) TPU sheets were cut into grid-like patterns, followed by spray-coating nanocomposite paint to form sensing mesh specimens. Then, the sensing mesh specimens were affixed onto the PVC plate. (**b**) The sensing mesh was affixed onto an intact PVC plate for strain distribution monitoring, along with a strain gage mounted on the backside. (**c**) The sensing mesh was affixed onto a cracked PVC plate for crack detection tests.

As mentioned in previous sections, strain gages can only measure strain at a point. This study aimed to design a strain sensing mesh that could reconstruct the strain distribution over its applied surface area. A piece of TPU sheet (Figure 3a) was laser-cut (Silhouette Cameo 3) into a grid-like pattern. A layer of nanocomposite paint was then spray-coated onto the TPU pattern to form a sensing mesh specimen. Twelve equidistantly spaced boundary electrodes were applied along the boundary of the sensing mesh using two-part silver epoxy and single-strand electrical wire. The silver epoxy is a cold soldering material that could form conductive seals to fix the wire at its desired location. Two sets of sensing mesh tests were conducted as described below.

#### 3.3.1. Distributed Strain Monitoring

**Figure 4:**
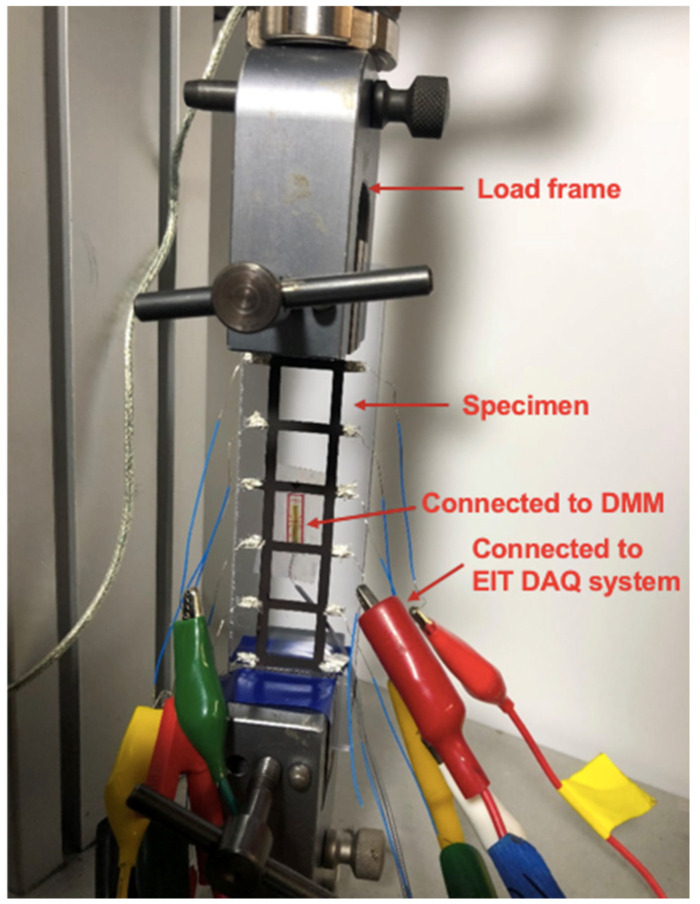
The entire specimen was mounted in the load frame and subjected to uniaxial tensile loading. ERT measurements were collected at different loading states and then used as the inputs of ERT conductivity reconstruction analysis. The magnitude of strain induced in the specimen was recorded by the strain gage.

The sensing mesh specimen was affixed onto an intact 35 × 150 mm^2^ clear polyvinyl chloride (PVC) plate that was 1/8” thick (Figure 3b). A strain gage was mounted on the back of the PVC plate to measure longitudinal strains in the loading direction. The PVC plate was then mounted onto the load frame (Figure 4) for distributed strain sensing tests. The load frame was programmed to apply tension to a total displacement of 0.6 mm at a load rate of 0.05 mm/s, pausing every 0.2 mm for ERT data collection using a customized data acquisition (DAQ) system. Here, a Keithley 6221 AC/DC current generator was used to input DC current excitations, and it was interfaced with a Keysight 34980A multifunctional switch with an embedded DMM for voltage measurements. A MATLAB script controlled the switch and current generator, enabling automated voltage data acquisition based on a predefined, multi-path, current injection pattern. The adjacent current injection pattern (i.e., the current was injected to adjacent electrode pairs) was used in this study, and the corresponding boundary voltage measurements for each current injection path were collected during testing [18]. Prior to applying the load, a baseline ERT dataset was recorded when the specimen was unloaded. Strain gage data were also recorded throughout the entire tests.

#### 3.3.2. Sensing Mesh Crack Identification

In order to further demonstrate the sensing mesh’s ability to map the physical properties of damage, the sensing mesh was applied onto another set of 35 × 150 mm^2^ PVC plates with a center crack (Figure 3c). The specimen was subjected to uniaxial tensile loads at 0.05 mm/s up to a maximum displacement of 0.4 mm. Similar to previous experiments in Section 3.3.1, a baseline ERT measurement was recorded when the specimen was unstrained. ERT measurements were collected when the load frame was paused after the crack propagated.

## 4. Results and Discussion

### 4.1. Nanocomposite Paint Formulations

**Figure 5:**
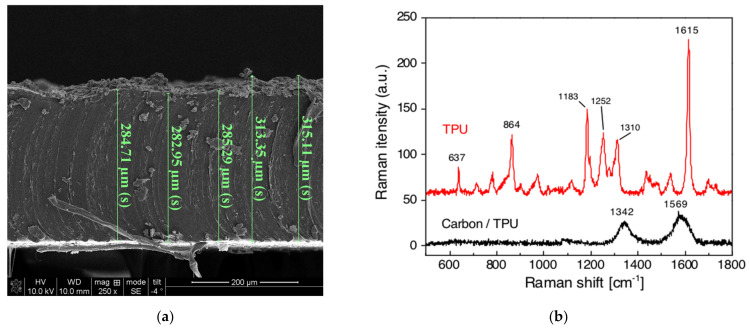
(**a**) A scanning electron micrograph of the nanocomposite paint cross-section was obtained to estimate its thickness and uniformity. (**b**) Raman spectra of the nanocomposite paint (in black), as well as the TPU substrate (in red), are shown.

Nanocomposite paint specimens were fabricated according to the procedure discussed in Section 3.1. Scanning electron microscopy (SEM) was conducted to examine the uniformity of the as-deposited paint. Figure 5a shows that the thickness of the paint was ~30 µm and uniform over the measured region. In addition, Raman spectroscopy was performed. Figure 5b shows the Raman spectra of the TPU substrate and nanocomposite paint deposited on the same TPU. The Raman spectrum of TPU substrate shows the major Raman peaks of polyurethane, including the vibrational mode of aromatic rings (637 cm^−1^), out-of-plane bending of aromatic C-H (864 cm^−1^), C-O stretching of the alcohol (1183 cm^−1^) and the ester group (1252 cm^−1^), and C=C bonds in the aromatic rings (1615 cm^−1^) [36]. In contrast, the Raman spectrum of the nanocomposite paint on TPU does not exhibit TPU Raman peaks, while the D- (1342 cm^−1^) and G-bands (1569 cm^−1^) of carbon nanotubes were clearly identified [37]. These Raman analysis results confirm that MWCNTs were successfully embedded in the paint matrix and coated onto the TPU surface.

**Figure 6:**
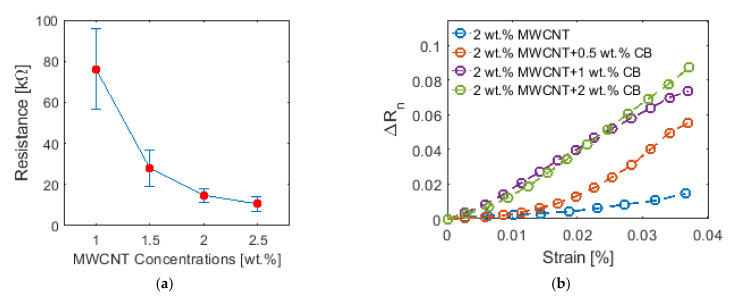
(**a**) The average electrical resistance of different MWCNT concentration nanocomposite paint specimens and their corresponding error bars (standard deviations) are plotted. (**b**) The normalized change in resistance of different paint formulations were plotted against applied strains to examine strain sensing linearity and the effects of adding CB.

The influence of MWCNT concentration on the conductivity of the nanocomposite paint was characterized. For each of the four different MWCNT concentrations (i.e., 1, 1.5, 2, and 2.5 wt.%), six different specimens were tested. Figure 6a plots the average electrical resistance with respect to MWCNT concentrations. The error bars indicate the standard deviation. It can be seen that coatings with higher MWCNT concentrations tended to have lower resistance (i.e., higher conductivity). This can be explained by the fact that MWCNTs form electrical pathways for current to pass through. Therefore, higher MWCNT concentrations led to higher conductivity coatings. It was also observed that the rate of resistance decrease slowed as the MWCNT concentration was increased, exhibiting an exponential decay trend. Since the electrical conductivity change was minor when the MWCNT concentration was increased from 2 to 2.5 wt.%, a paint formula with 2 wt.% MWCNT was selected and used for the strain sensitivity characterization tests.

The nanocomposite’s strain sensitivity was characterized using data from the cyclic tensile tests as described in Section 3.2. The normalized change in resistance (∆*R_n_*) was calculated using the difference between the gage resistance at a particular strain state (*R_i_*) and its unloaded state (*R*_0_), and then normalized by *R*_0_:

(10) $$\Delta R_{n}=\frac{\Delta R}{R_{0}}=\frac{R_{i}-R_{0}}{R_{0}}\;$$

As shown in Figure 6b, a linear relationship was not observed between ∆*R_n_* with respect to the applied strains for the 2 wt.% MWCNT nanocomposite paint. To further improve its linearity, different concentrations of carbon black (CB) ranging from 0.5 to 2 wt.% were added to modify the paint formulation. As seen in Figure 6b, adding CB to the nanocomposite paint not only improved the sensor linearity but also increased the strain sensitivity. Since the difference in strain sensitivity between paints with 1 wt.% CB and 2 wt.% CB was negligible, the optimum paint formulation was determined to be with 2 wt.% MWCNT and 1 wt.% CB. Thus, the final nanocomposite paint formulation consisted of 37 wt.% of vinyl paint, 61.89 wt.% of vinyl paint thinner, 0.74 wt.% of MWCNT, and 0.37 wt.% of CB.

### 4.2. Nanocomposite Paint Strain Sensing Properties

**Figure 7:**
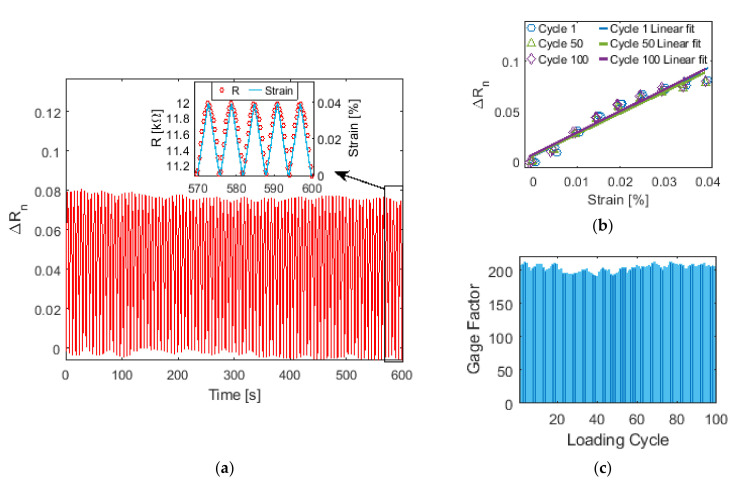
(**a**) The normalized electrical resistance time history of a nanocomposite thin film subjected to 100 tension cycles are plotted. The inset shows the response for the last five loading cycles and is overlaid with the applied strains. (**b**) Sensor hysteresis was examined by considering the resistance change for three different loading cycles with respect to the applied strains. (**c**) The gage factor for each of the 100 loading cycles are plotted.

Upon determining the optimal paint formulation for this study, uniaxial tensile cyclic load tests were performed to characterize the electromechanical properties of the nanocomposite coating. Figure 7a shows a representative ∆*R_n_* time history plot from a 100-cycle tensile. The resistance response of the last five cycles, along with the applied strain pattern, were also zoomed in and are shown as an inset of Figure 7a. This result confirmed that the resistivity of the nanocomposite coating varied in tandem with applied strains and remained stable and repeatable over multiple loading cycles.

Furthermore, as shown in Figure 7b, the same set of data was used to plot its hysteresis response. Here, three different loading cycles were randomly selected and plotted. Barely any hysteresis response was observed. Linear least-squares regression lines were also fitted to the data from each loading cycle, and strain sensitivity or gage factor (*G*) was defined as the slope of the best-fit line:

(11) $$G=\frac{\Delta R_{n}}{\Delta \varepsilon }=\frac{\Delta R/R_{0}}{\Delta \varepsilon }\;$$

where Δ*ε* is the change in applied strains. The gage factors for all 100 loading cycles are plotted in Figure 7c. The standard deviation of *G* was only 5.49, while the average *G* was found to be 203, which is ~96-times higher than that of conventional strain gages. Moreover, it was also higher than other published results of spray-fabricated nanocomposite thin films [38,39]. Therefore, the proposed nanocomposite paint possesses desirable strain sensing properties for various engineering applications.

### 4.3. Sensing Mesh for Distributed Strain Monitoring

**Figure 8:**
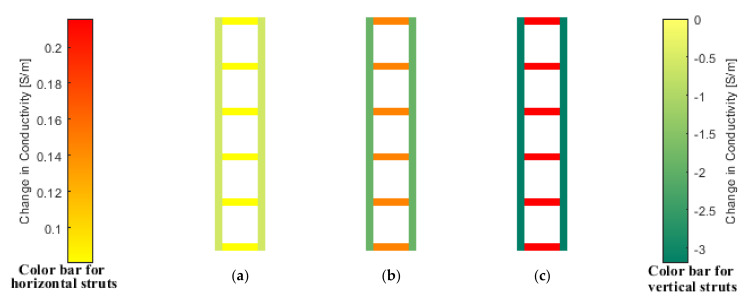
The reconstructed ERT conductivity maps when the sensing mesh was subjected to uniaxial tensile loads when the vertical displacement of the load frame grips was (**a**) 0.2, (**b**) 0.4, and (**c**) 0.6 mm are shown. Two color bars are used to visualize conductivity changes the for horizontal (**left** color bar) and vertical (**right** color bar) struts.

Sensing meshes were formed by spray-coating nanocomposite paint onto grid-like patterned substrates and subjected to tensile tests as described in Section 3.3.1. Figure 8 shows a representative ERT result. The PVC plate was subjected to a total displacement of 0.2, 0.4, and 0.6 mm, respectively, in the longitudinal direction. It can be observed from Figure 8 that the conductivity changes of the longitudinal struts were negative, indicating an increase in resistance due to the existence of tensile strains. On the other hand, struts in the transverse direction increased in conductivity (or resistivity decreased) as a result of compressive strains caused by Poisson’s effect. The trend of the conductivity change of the sensing mesh was expected. The higher the total displacement applied, the higher the magnitude of the change in conductivity, as can be observed in Figure 8.

**Figure 9:**
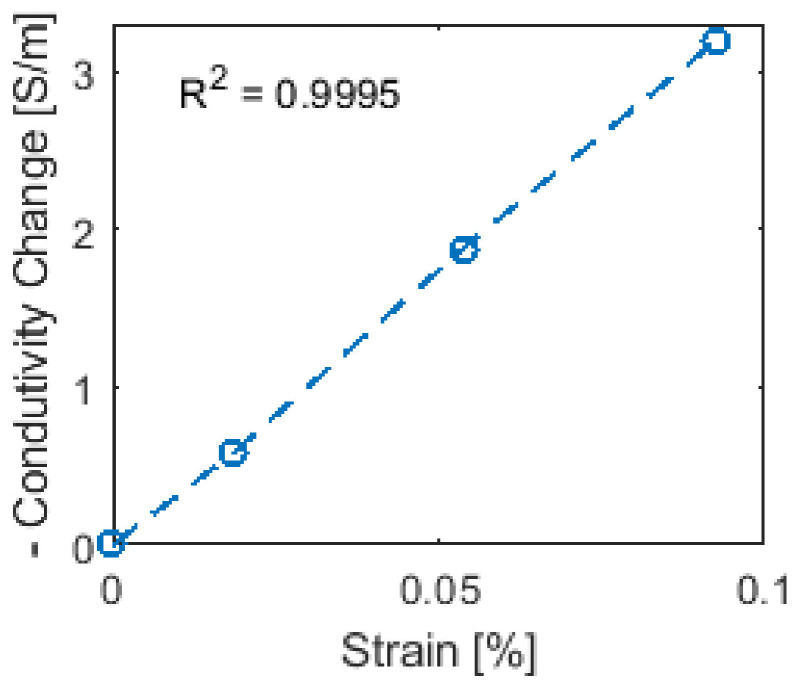
The vertical struts’ average conductivity change from sensing mesh ERT results were calculated and plotted against the strain gage measurements of the PVC plate. Conductivity decreased as greater tensile strains were applied.

To further investigate the accuracy of the ERT strain sensing results, the longitudinal strain developed in the PVC plate was measured by a strain gage on its backside. Assuming strains were uniformly distributed in the PVC plate during uniaxial loading, the conductivity changes of the sensing mesh’s longitudinal struts were averaged and plotted against the strain gage data as shown in Figure 9. From Figure 9, it can be seen that the change in conductivity of the longitudinal struts increased linearly as greater tensile strains were applied. A linear least-squares regression line was also fitted and is shown in Figure 9. The coefficient of determination (*R*^2^) of the regression line was found to be 0.9995, which indicates a strong linear relationship. Overall, the results shown in Figure 7, Figure 8 and Figure 9 confirmed the ability of the sensing mesh to accurately measure distributed strains.

### 4.4. Crack Monitoring

This study also investigated whether the sensing mesh could localize the presence of severe damage such as cracks. In general, cracks are highly localized damage features that are difficult to detect and quantify using a distributed network of strain gages unless the crack happens to occur at the location of a strain gage. However, at the location of the crack, a crack opening can be regarded as highly localized tensile strains in the direction perpendicular to the crack length. The tests described in Section 3.3.2 sought to validate whether sensing meshes could determine the location of a crack.

**Figure 10:**
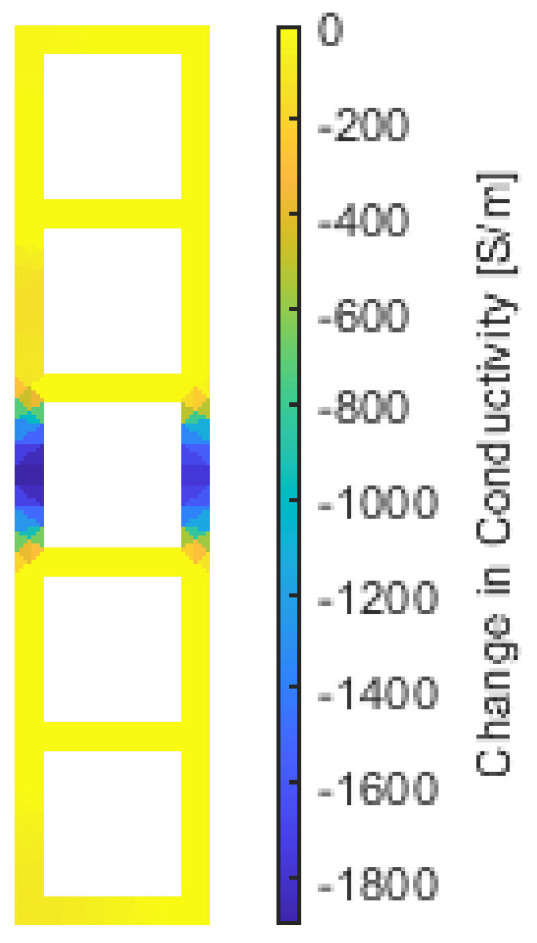
The change in conductivity distribution of the sensing mesh with respect to the baseline before tensile strain excitation. EIT results show the corresponding damage area when the vertical displacement of the load frame grips was loaded to 0.4 mm.

Figure 10 shows the reconstructed ERT result of a sensing mesh subjected to the crack propagation test. The ERT result clearly shows a highly localized decrease in conductivity where the crack was located (see Figure 3c). Although preliminary, these results confirm that the sensing mesh could identify the location of the crack to be within the region covered by the two struts with large magnitude conductivity decreases. To improve crack mapping resolution, one approach could be to increase the density of the sensing mesh grid pattern. However, a denser sensing mesh grid would require more boundary current interrogations and voltage measurements for accurate ERT conductivity distribution reconstruction.

## 5. Conclusions

In this study, a nanocomposite paint for distributed strain monitoring was developed. First, the nanocomposite paint formulation was optimized based on its conductivity and linearity. Second, its electromechanical properties were characterized via the cyclic tensile test. The results confirm its linear, repeatable, strain sensing response, and strain sensitivity was 203, or ~96-times higher than conventional strain gages. Third, sensing meshes were fabricated by spray-coating the nanocomposite paint onto a grid-patterned substrate. By coupling sensing meshes with an ERT measurement technique and algorithm, their conductivity distribution (which is correlated to strain) could be reconstructed. Tensile load tests confirmed distributed strain mapping, where longitudinal struts could measure the applied tensile strains, while the transverse struts measured compressive strains due to Poisson’s effect. Last, the sensing mesh was also mounted onto a test coupon, where a crack was propagated during loading. The ERT results confirmed localization of the crack within the grid-like sensing mesh. Overall, the experimental results validated the distributed strain sensing capability of the nanocomposite paint. Future research will focus on in situ field tests of the nanocomposite paint applied onto large structures.

## Data Availability

Not applicable.
